# Molecular and Neural Mechanism of Dysphagia Due to Cancer

**DOI:** 10.3390/ijms22137033

**Published:** 2021-06-29

**Authors:** Ikuko Okuni, Yuta Otsubo, Satoru Ebihara

**Affiliations:** Department of Rehabilitation Medicine, Toho University Graduate School of Medicine, Tokyo 143-8541, Japan; pon1990@med.toho-u.ac.jp (I.O.); yuta.otsubo@med.toho-u.ac.jp (Y.O.)

**Keywords:** taste, swallowing, cachexia, sarcopenia, inflammation

## Abstract

Cancer is one of the most common causes of death worldwide. Along with the advances in diagnostic technology achieved through industry–academia partnerships, the survival rate of cancer patients has improved dramatically through treatments that include surgery, radiation therapy, and pharmacotherapy. This has increased the population of cancer “survivors” and made cancer survivorship an important part of life for patients. The senses of taste and smell during swallowing and cachexia play important roles in dysphagia associated with nutritional disorders in cancer patients. Cancerous lesions in the brain can cause dysphagia. Taste and smell disorders that contribute to swallowing can worsen or develop because of pharmacotherapy or radiation therapy; metabolic or central nervous system damage due to cachexia, sarcopenia, or inflammation can also cause dysphagia. As the causes of eating disorders in cancer patients are complex and involve multiple factors, cancer patients require a multifaceted and long-term approach by the medical care team.

## 1. Introduction

### 1.1. Advances in Cancer Care and Cancer Survivorship

Cancer is a disease that affects many people and is one of the leading causes of death worldwide. While the number of cancer cases is increasing in Japan’s rapidly aging society, the cancer survival rate is also improving every year because of advances in diagnostic and therapeutic techniques. Since 1981, cancer has been the leading cause of death in Japan, accounting for approximately 30% of deaths [1]. A Japanese individual’s cumulative risk of cancer in their lifetime is 65.5% for men and 50.2% for women. Epidemiological research has shown that one in two people experience some form of cancer [2]. Nevertheless, the age-adjusted mortality rate for cancer has been declining since the 1990s, resulting in a growing population of cancer “survivors” [3]. There are still several challenges with medical therapies for cancer patients. Therapeutic advancements mean that we may be approaching a time when cancer will not be an incurable disease but rather a chronic condition, such as hypertension or diabetes. In 1985, a doctor diagnosed with mediastinal germinoma published an essay about his experience battling the disease that stressed the importance of the treatment process in cancer patients after diagnosis [4]. Cancer survivorship encompasses the entire life after cancer diagnosis and treatment. While conventional cancer research has focused on the periods immediately after cancer diagnosis, during treatment, and close to death, cancer survivorship research assumes that patients will continue to live active social lives after the initial treatment and is characterized by addressing the various challenges faced by the patients and their families in their everyday lives [5].

The issues addressed by survivorship research in Japan include (1) dealing with long-term complications and screening for delayed effects and secondary cancers: incidence and associated factors, economically efficient follow-up methods, and a collaboration between specialists and family physicians; (2) relationships: couples, parents and children, siblings, friends, and coworkers, etc.; (3) lifestyle and health promotion: smoking, alcohol consumption, exercise, diet, and sleep; (4) life course-specific issues: love, marriage, sex, having children, childcare, and nursing care; (5) school and work problems; (6) economic problems: medical expenses, living expenses, or various forms of insurance, etc.; (7) cancer rehabilitation; and (8) existential problems related to being alive [6,7]. Within the field of survivorship research, studies of lifestyle improvements and health promotion through diet and other means have attracted attention because these are areas that patients and their families can control [8].

Medical care aims at improving the functioning and quality of life of cancer patients to maximize their physical, social, and psychological activities within the limits imposed by the cancer and its treatment. Disability in cancer patients can be caused by various factors. Therefore, continuous uninterrupted care is required. It begins with the prevention of functional decline and complications from the time of diagnosis or prevention and aims at early recovery after treatment. Then, it continues from the perioperative period and chemotherapy/radiation therapy aiming at radical cure to care for patients with advanced cancer and terminal cancer. In order to provide appropriate medical care to patients, it is important to raise awareness, share information, collaborate with various specialists, and carry out a comprehensive care process from the time of diagnosis.

### 1.2. Swallowing Function in Cancer Patients

The recognition of food, intake of food into the oral cavity, and swallowing of food and liquid along with saliva is a complex process of biomechanical interactions involving physiology and anatomy. It consists of five phases: pre-oral, oral preparatory, oral transit, pharyngeal, and esophageal. Swallowing involves the central nervous system (CNS), muscles, and the cranial nerves connected to them. Neurological or structural defects can affect swallowing, which can cause dysphagia. Dysphagia is a common complication in cancer patients and is associated with several factors, including the direct effects of tumors, resection of the cancer, chemotherapy, and radiation therapy. Although these complications are often studied in patients with head and neck cancer, dysphagia can also occur in other cancers [9].

Frowen et al. investigated the prevalence of dysphagia in patients (*n* = 239) being treated for cancer (chemotherapy, radiation therapy, and/or surgery), except those receiving end-of-life care. Patient-reported dysphagia was observed in more than half of the patients (54.4%). By cancer type, the prevalence of dysphagia was highest in head and neck cancer (89%). A high rate was also reported by patients presenting with all tumor types: lung (78%), bone and soft tissue (73%), upper gastrointestinal (67%), colorectal (62%), skin/melanoma (55%), gynecologic (42%), and breast cancer (32%). They also investigated the oral complications across all cancer types and noted that taste change (62%) had the highest prevalence [10]. The prevalence of swallowing difficulties at the end of the palliative phase in cancer patients has been reported to be 58–77% according to a questionnaire [11].

The causes of eating disorders in patients with cancer are complex and involve multiple factors. Initially, it is primary anorexia (that is, cause at CNS level), which can be exacerbated by secondary disorders involving oral feeding. The main secondary causes of reduced intake include oral ulcers, dry mouth, poor tooth quality, intestinal obstruction, malabsorption, constipation, diarrhea, nausea, vomiting, reduced intestinal motility, changes in the senses of taste and smell, fatigue, cancer pain, and psychological distress, several of which affect the swallowing functions [12,13,14].

Dysphagia has a significant impact on the quality of life in patients, compromises vital functions, such as eating, and can be potentially life-threatening. It also has a great impact on the quality of life of the caregivers. The pathophysiology of dysphagia is quite complex and variable throughout the course of the disease, and multiple mechanisms may overlap. Recognizing the onset of dysphagia and implementing interventions to prevent or mitigate its effects are essential for optimal patient management. In addition, cancer patients require a multifaceted and long-term approach by the medical care team.

This paper outlines CNS-related dysphagia, taste and smell disorders, and dyspha-gia due to loss of skeletal muscle.

## 2. Dysphagia in Cancer Patients

### 2.1. CNS and Swallowing

#### 2.1.1. CNS Control of Swallowing

Swallowing is a complex combination of movements achieved by voluntary (CNS) and involuntary reflexes. The swallowing process activates a network of regions in the brain, including the bilateral sensory-motor cortex, primary motor area, premotor area, supplementary motor area, insular cortex, cingulate gyrus, inferior frontal gyrus, inferior parietal lobule, temporal lobe, precuneus, basal ganglia, cerebellum, and brainstem [15,16]. It also requires a highly organized regulatory system. A swallowing central pattern generator (CPG) in the medulla oblongata has been found to play a major role. The input–output loop of swallowing movements through the medulla oblongata is thought to be mediated by the swallowing CPG [17]. As swallowing requires a large neural network, dysphagia can occur when there is a cancer lesion in the brain.

The swallowing CPG is located in the nucleus tractus solitarii (NTS) and the ventrolateral medulla near the nucleus ambiguus (NA) [17]. It consists of four units, two on each side, which receive both input and output information that is used to adjust to each phase of swallowing. The NTS receives information from the NA, which sends commands to the muscle tissues that are most important for swallowing, via motor neurons. The NTS also receives input from the oral cavity, pharynx, laryngeal mucosa, and cerebral cortex to regulate the initiation and continuity of swallowing based on the characteristics of the bolus, such as its size, texture, and temperature. Cancer lesions can cause dysphagia by interrupting these connections. In addition, the two swallowing CPGs are tightly synchronized and control the coordinated contractions of the bilateral muscles of the oropharyngeal region. An anatomical connection mediated by fibers that cross the midline exists between the two swallowing CPGs [18].

At present, there are only hypotheses regarding the mechanisms by which the swallowing CPGs process information that is expressed in the movements of swallowing. Analyzing the neuron types in neural circuits is important for verifying the information processing mechanism. Regarding the neurotransmitters of the neurons that make up the swallowing CPGs, excitation amino acid receptors, mainly of the N-methyl-D-aspartic acid type, are reported to be involved in generating the swallowing motion and creating continuous movements in the NTS. Moreover, the inhibitory mechanism has been reported to be a gamma aminobutyric acid (GABA)-related mechanism with cholinergic neurons associated with pharyngeal-esophageal movements [17,19].

#### 2.1.2. Coordination of Swallowing and Breathing

Research on swallowing function has mainly focused on physiological and neuroanatomical aspects, as well as on their association with respiration. Since swallowing and breathing share muscles and anatomical structures, these two functions need to be highly coordinated. A respiratory CPG controls the respiratory movements in the brainstem. There is a dorsal respiratory group on the ventrolateral side of the NTS in the medulla oblongata, and a caudoventral respiratory group, rostroventral respiratory group, pre-Bötzinger complex, Bötzinger complex, and parafacial respiratory group on the ventrolateral side of the medulla oblongata. There is also a pontine respiratory group in the dorsolateral region of the pons. These neuron groups work closely together [20]. There are several swallowing patterns with coordinated breathing and swallowing; however, for most single swallows, respiration stops during exhalation when a swallow occurs and then resumes with exhalation. This exhalation–swallowing–exhalation pattern is best for preventing the pharyngeal contents from entering the lower respiratory tract, as aspiration often occurs during inhalation [21,22,23]. Martin-Harris et al. reported that bio-feedback approaches can improve the swallowing function in head and neck cancer patients with postoperative disharmony between respiration and swallowing [24].

As for the neural mechanisms, studies on transgenic mice with channelrhodopsin-2 or archaerhodopsin specifically expressed in glycinergic neurons have concluded that the activation of glycinergic neurons in the pre-Bötzinger complex may reset the respiratory cycle and that the activation of glycinergic neurons in the rostroventral respiratory group may have a major impact on respiratory patterns [25,26,27]. The timing of swallowing also depends on feedback from the lungs, such as the lung volume, suggesting that the respiratory and swallowing CPGs exert control by interacting and sharing several mechanisms, including short-term plasticity. This phenomenon may be of interest in the rehabilitation of patients with cancer with dysphagia [21,28]. On the contrary, the activation of an inflammatory response may impact areas of the CNS, such as the basal ganglia (especially the ventral corpus striatum), dorsal anterior cingulate cortex, dorsolateral prefrontal cortex, amygdala, hippocampus, insular cortex, caudate nuclei, and putamen in cancer patients with systemic inflammatory diseases [29,30]. It has been reported that a latent reduction in the release of GABA and acetylcholine in the brain in response to inflammatory cytokines promotes inflammatory responses [29]. Since cancer patients are at risk of chronic undernourishment and dysphagia after developing cancer, a multidisciplinary approach to management is needed based on a good understanding of this mechanism.

### 2.2. Important Roles of Taste and Smell in Swallowing

Taste and smell disorders in patients with cancer are very common. They occur not only as symptoms of the disease in patients with head and neck cancer, but are often overlooked side effects of chemotherapy. If not dealt with properly, they can affect the nutritional status, food enjoyment ability, and quality of life [31,32]. The prevalence of dysgeusia during cancer therapy ranges from 20 to 86%; additionally, changes in smell are found in 5–60% of cases [33,34]. The root cause of these conditions remains unknown. Changes in taste and smell have also been reported in nearly half of the cancer patients with no history of treatment, who do not have solid cancers of the head and neck. This is approximately 100 times the prevalence of taste and smell disorders in the general population. Treatment can exacerbate taste and smell disorders, which can lead to poor nutritional status [35,36]. Furthermore, 86% of patients with advanced cancer receiving palliative care had taste and/or smell disorders, while 52% had both taste and smell disorders. In severe cases, these are thought to be major contributors to malnutrition and wasting [37].

In the oral cavity, taste is sensed mainly by gustatory cells in the taste buds of the tongue. These have taste receptors on their surfaces that bind to tastants. The tastants of sweet, bitter, and umami mainly bind to G protein-coupled taste receptors, while salty and sour tastants bind to ion-channel taste receptors (Table 1) [38,39]. The five basic taste receptors receive the five basic tastes and are transmitted to the NTS in the medulla oblongata via the gustatory nerves (the chorda tympani, lingual branch of the glossopharyngeal nerve). Subsequently, the taste input is transmitted to areas, such as the gustatory cortex of the cerebral cortex and motor cortex, which promote the secretion of saliva and gastric juices and distinguish flavor, and to the hypothalamus and amygdaloid nucleus, which control the emotional system. These factors are thought to modulate voluntary swallowing and eating. Additionally, the senses of smell and taste are integrated in the cerebral cortex to produce the flavor of food [40,41,42]. Therefore, dysgeusia is believed to be associated with abnormalities in the taste reception mechanism from taste reception to perception in the CNS. Moreover, taste buds are present in the mucous membranes of the pharynx and larynx. Taste and other sensory inputs received via the pharyngeal and laryngeal mucosa are sent to the swallowing center via the pharyngeal branch of the glossopharyngeal and superior laryngeal nerves. This information is processed by the swallowing center, which modulates swallowing [43,44,45]. Chemosensory ion channels are activated along with gustatory receptors when chemical stimulation is applied to swallowing-related regions. Temperature-sensitive transient receptor potential (TRP) channel families (TRPV1, TRPA1, and TRPM8) and acid-sensing ion channels have been reported. For elderly patients and those with stroke and neurodegenerative disease with oropharyngeal dysphagia, ion channel agonist supplementation provides evidence of neural network plasticity [46,47,48].

Humans sense smell via two pathways: the oronasal pathway, in which the aroma components pass through the anterior nostrils into the nasal cavity, and the retronasal pathway, in which they travel from the oral cavity into the nasal cavity via the choanae [49]. Although there is disagreement as to whether these two pathways demonstrate “duality in the sense of smell” via different neural circuits [50,51,52], there is agreement that the sense of smell—especially the retronasal sense in which smells, dispersed into the air in the oral cavity during chewing and swallowing, pass from the oral cavity to the pharynx and nasal cavity via exhalation—shares neural circuits with the sense of taste [53,54]. Furthermore, the gustatory cortex and the olfactory (piriform) cortex integrate information, suggesting that they are involved in multimodal information processing [55,56]. In addition, dysgeusia, commonly seen in cancer patients undergoing chemotherapy, manifests as bitter, metallic, salty, or unpleasant tastes and is closely related to changes in the sense of smell [57,58]. Furthermore, the integration of taste and smell information is essential for perceiving taste, and swallowing depends on the interaction between the taste and smell systems.

#### 2.2.1. Drug-Induced Taste Impairment

Table 2 shows the antineoplastic agents that can cause dysgeusia. Dysgeusia is more common with drugs such as antimetabolites, taxanes, and molecular-targeted agents. Amézaga et al. [58] conducted a prospective observational study using a questionnaire that included items on the changes in taste and smell. They reported that the taste change scores were highest in patients taking docetaxel, followed by carboplatin, anthracycline, paclitaxel, and others. High scores in smell change were observed with vinorelbine and anthracycline. They also examined changes in the sense of smell and pointed out that smell changes should also be considered in dysgeusia caused by antineoplastic agents. The mechanism by which antineoplastic agents change taste and smell is still not understood. It probably involves numerous factors, including mucitis, zinc depletion, changes in gustatory cells, changes in saliva volume and composition, disruption of receptor activity, increased inflammation, intraoral diffusion of drug components, and inhibition of receptor cell replication [32,34]. Even deep underlying mechanisms have been proposed, including damage to the cranial nerves (for example, demyelination of nerve fibers), tissue necrosis and infection, nasal obstruction, modification of the afferent pathways by which antineoplastic agents pass through the blood-brain barrier, and drug-induced neuropathy and neurotoxic effects [34]. Tsutsumi et al. [59] investigated the changes in the gene expression of taste receptors in the lingual mucosa of patients with head and neck cancer who were administered fluorouracil and cisplatin. They found a reduced expression of T1R3, a subunit common to umami and sweetness receptors, and an increased expression of T2R5, a bitterness receptor. There was also a report of increased expression of T1R2, a sweetness receptor subunit, in the circumvallate papillae of rats administered oxaliplatin [60]. van der Werf et al. [61] reported that changes in the sensitivity to bitterness in patients with metastatic cancer during the administration of protein kinase inhibitor molecular-target drugs may be explained by interactions with G protein-coupled taste receptors.

#### 2.2.2. Radiation-Induced Taste and Smell Impairment

In cancer treatment, radiation therapy is often performed in combination with chemotherapy. In addition to being a radical therapy and helping to prevent recurrence, radiation therapy may be used for pain relief in advanced cancers. Various adverse reactions may occur during radiation therapy, depending on the irradiation site. Disorders of the oral mucosa and dysgeusia are particularly common in patients with head and neck cancer, as the irradiation site includes the tongue and oral cavity. Changes in taste are mainly due to damage to gustatory cells from radiation therapy; however, as radiation therapy often affects the amount and composition of saliva, dry mouth (xerostomia) can also contribute to changes in taste. The pattern of dysgeusia is greatly influenced by the distribution of taste buds that are damaged during radiotherapy. It is also known that the severity of dysgeusia is proportional to the radiation dose to the tongue [40]. Disorders of the five basic tastes can appear from the start of radiation therapy to a few weeks later, with bitter and salty being the most impaired, and sweetness the least. The loss of the four basic tastes has not been reported up to 20 Gy of radiation to the head and neck region; however, it increases exponentially up to a cumulative dose of 30 Gy, with a relative taste loss seen in more than 90% of patients at 60 Gy [62,63]. The disorders of umami have a unique pattern, increasing after a threshold of 15 Gy with significant impairment at 30 Gy [64].

Dysgeusia gradually recovered after the end of radiation therapy. Approximately six months after the end of radiation therapy, improvement is seen in discomfort, dysgeusia, and general taste changes; however, some loss of basic taste remains. Some loss of all the tastes has been reported 1–2 years after treatment, with some patients experiencing permanent taste decline [63,65].

Several models have been proposed to explain the mechanism of dysgeusia in radiation therapy. Notably, recent research has focused on gustatory cells. Radiation primarily targets proliferating cells, inducing a dramatic decrease in precursor cells and a significant increase in apoptosis in precursor cells. As a result, there is an insufficient supply of new cells; when the natural loss of differentiated (old) gustatory cells is not immediately replaced, there is a decrease in the functional gustatory cells, resulting in dysgeusia [66,67]. This suppression of growth involves the downregulation of the Wnt/β-catenin pathway [68]. In a study on reducing dysgeusia, Qiang Guo et al. [69] reported that sirtuin-1 inhibitors in taste bud organoids promoted taste stem cell survival after irradiation. Similarly, Yuan et al. [70] reported that irradiation-induced dysgeusia was reduced by inhibiting apoptosis via p53 in gustatory cells in checkpoint kinase 2 knockout mice.

Dysosmia caused by radiation therapy for head and neck cancer has been less studied than dysgeusia. A systematic review of the studies of dysosmia due to radiation therapy found that the thresholds for smell detection and identification were impaired immediately after the completion of radiation therapy and were bad in patients who received simultaneous chemotherapy. Recovery from dysosmia generally took six months after treatment; however, it could take an additional 20 months if the olfactory epithelium received a dose exceeding 10 Gy. While smell identification recovers, in some patients, problems with smell detection have been observed up to five years after radiation therapy [71]. Studies on the mechanism of dysosmia have observed suppressed growth of olfactory stem cells in the olfactory nerve epithelium after irradiation and a halt in mitosis in the basement membrane of the olfactory epithelium. There was no difference in apoptosis in the olfactory epithelium between the irradiation and control groups. Reduced neurogenesis, small olfactory bulbs, changes in the subtypes of the interneurons connecting the olfactory epithelium to the olfactory bulbs, and a significant reduction in GABAergic subtypes have been observed [72,73].

Radiation destroys tissue homeostasis by damaging the DNA in the nuclei of rapidly proliferating cells (tumors, epithelium, etc.) and hindering the normal functioning of organelles in the surrounding cells. Therefore, radiation therapy for head and neck cancer causes collateral damage to adjacent healthy tissues, which can lead to acute dysphagia. Chronic dysphagia can be caused by muscle atrophy (radiation-induced fibrosis) in the larynx and pharynx, and radiation-induced neuropathy in the peripheral and cranial nerves that govern the swallowing muscle system. These are mainly caused by the activation of transforming growth factor β, although the mechanism is not well understood. Muscle atrophy may also result from the disuse of the oropharyngeal muscle system; thus, it is considered important to address any taste and olfactory disorders that occur before or during radiation therapy [74,75,76].

#### 2.2.3. Management of Taste and Smell Impairment in Cancer

Pharmacological management strategies have been investigated to determine the efficacy of zinc supplementation in alleviating taste changes in patients with cancer. A recent systematic review indicated that zinc may have a positive effect on taste changes in cancer patients [77]. The efficacy of polaprezinc (zinc L-carnosine), a gastric mucosal protector, has been shown to resolve taste changes [78,79,80]. The use of medical cannabis, especially delta-9-tetrahydrocannabinol, can be expected to significantly reduce subjective taste and smell disturbances from baseline [81]. Many of the effects of the chemotherapeutic drug cyclophosphamide on the taste system due to its cytotoxicity can be reduced by pretreatment with amifostine [82]. In addition, cyclophosphamide can affect cell regeneration in the olfactory system, which may contribute to clinical loss of function during and after chemotherapy [83]. Therefore, amifostine may reduce taste and smell disorders. The neuroprotective effects of glutamine were studied by Strasser et al. [84]. under the hypothesis that taste disorders caused by taxane chemotherapy are a consequence of neurological damage. Glutamine had no preventive effects on taste disorders.

There have been several studies on non-pharmacological management strategies, and in a case report by El Mobadder et al. [85], photobiomodulation therapy was considered an effective approach for the management of taste alterations in cancer patients. In a case study on acupuncture, the authors concluded that acupuncture had a protective effect on nerve tissue and played a role in reducing the complaints of dysgeusia and odynophagia caused by radiotherapy [86].

Oral cryotherapy is thought to be effective in reducing local exposure to anticancer drugs by narrowing the blood vessels in the oral cavity [87]. Taste disturbances caused by anticancer drugs (docetaxel, cisplatin, and 5-fluorouracil) may be partially prevented by cooling the tongue with ice water [88].

Prophylactic exercise therapy was investigated by Carnaby-Mann et al. [89]. The authors concluded that a prophylactic swallowing program can optimize functional outcomes in patients with head and neck cancer. Olfactory rehabilitation (nasal airflow-inducing maneuver) in patients with laryngeal cancer has been shown to have the potential to restore the sense of smell, regardless of the time elapsed after laryngectomy [90,91].

Self-care is important for minimizing the effects of taste and smell disorders. Various actions to cope with changes in taste include increasing or decreasing seasonings, eating lightly flavored foods, avoiding spicy foods, sucking on hard candy, eating small frequent meals, and taking care of the mouth [92,93]. For at-risk patients, counseling prior to chemotherapy or radiation therapy may not only reduce the incidence of taste disorders during treatment, but may also influence long-term taste disorders and improve the quality of life [94,95]. A recent study by von Grundherr et al. [96] concluded that enhanced nutritional counseling with taste and smell training may improve taste perception in patients undergoing chemotherapy.

Currently, there are several approaches for the prevention of taste and smell disorders. Combining these factors will bring increased benefits to patients with cancer. This requires a multidisciplinary team approach with the patients and caregivers at the center.

### 2.3. Dysphagia Due to Malnutrition and Loss of Skeletal Muscle Mass

#### 2.3.1. Cancer Cachexia

The term cachexia comes from the Greek words kakos (meaning “bad”) and hexus (meaning “condition”) and is used to describe the wasting syndrome seen in patients with chronic diseases, such as cancer. Researchers have proposed various definitions of cachexia in cancer. The current definition adopted by an international group of experts is “a multifactorial syndrome defined by an ongoing loss of skeletal muscle mass (with or without loss of fat mass) that can be partially but not entirely reversed by conventional nutritional support.” This group has also established diagnostic criteria and stage classifications [97]. Cachexia is observed in 50–80% of cancer patients and is estimated to account for 20% of cancer deaths [98]. It increases the susceptibility to treatment-related toxicity, lowers the quality of life, and increases functional disorders and cancer-related mortality [97]. It has been reported that starvation mainly reduces fat and is not accompanied by a significant loss of skeletal muscle; however, a major difference in cancer cachexia is that it involves a significant loss of both fat and skeletal muscle and induces substantial changes in the metabolism of glucose, lipids, and proteins [99].

With the goal of properly treating patients with cachexia, a classification system has been proposed that subdivides cachexia into three clinical stages: pre-cachexia, cachexia, and refractory cachexia. Patients with anorexia, impaired glucose tolerance, and weight loss of 5% or less in the last six months are classified as having precachexia. Patients with systemic inflammation, body mass index < 20, weight loss > 5% over the last six months, and ongoing weight loss greater than 2% are classified as having cachexia. Precachexia and cachexia require early multidisciplinary treatment [97]. Ideally, precancerous cachexia, and even the early stages require multimodal management.

#### 2.3.2. Mechanisms of Cachexia

The mechanism of the loss of muscle mass in cancer cachexia involves multiple factors and is still not fully understood. Multiple molecular pathways have been identified, in which the synergistic actions of several cytokines and other mediators eventually cause degeneration and necrosis of fat and muscle cells through signal transduction systems. These include the following: the (1) autophagic-lysosomal pathway, (2) insulin-like growth factor-1 pathway, (3) dystrophin-glycoprotein complex, (4) calcium-dependent proteolysis system, (5) mitogen-activated protein kinases, (6) interleukin-6, Janus kinase/ signal transducers and activators of transcription pathway, (7) myostatin/activin pathway, (8) poly (adenosine diphosphate-ribose) polymerase, (9) nuclear factor kappa-light-chain-enhancer of activated B cells-dependent pathway (including the tumor necrosis factor-related weak inducer of apoptosis, and the (10) ubiquitin-proteasome pathway. In addition, with reactive oxygen species and oxidative stress, the invasion of cancer cells into the adipose tissue induces the release of free fatty acids from the adjacent adipocytes, causing muscle wasting. Adipose triglyceride lipase and hormone-sensitive lipase are important enzymes that produce free fatty acids from adipose cells, and these enzyme systems are also involved in muscle deterioration [100,101,102].

Cancer cachexia is a syndrome that involves multiple factors, the development of which is governed and caused by inflammation. Skeletal muscle plays a central role in this syndrome, with various organs contributing to cancer cachexia, including the liver, heart, intestines, bones, adipose tissue, and the nervous system [103]. Cancer cachexia often involves a significant reduction in white adipocytes, which are a primary energy reserve. In addition, brown adipocytes with uncoupling protein 1 expression are observed inside the white adipose tissue, which increases lipid metabolism and heat production. This cell conversion is caused by the action of parathyroid hormone-related peptides, which are derived from the action of inflammatory cytokines and tumors. Adipose cell–muscle cell, adipose cell–cancer cell, and adipose cell–inflammatory cell crosstalk affects metabolic homeostasis [104]. Similar to adipose tissue and skeletal muscle, the liver plays an important role in controlling systemic metabolism. The liver takes lactic acid released by the skeletal muscles and converts it into glucose through gluconeogenesis, which is supplied to peripheral areas. In cancer cachexia, in addition to lactic acid from tumors, the liver uses substances, such as amino acids from the breakdown of muscle proteins and glycerol from lipolysis of adipose tissue for gluconeogenesis. The released glucose is consumed by advanced cancer cells in the glycolytic pathway and is thought to constitute a futile cycle that contributes to energy consumption [105].

#### 2.3.3. Poor Appetite and Loss of Skeletal Muscle Mass

Previous studies on loss of skeletal muscle in cancer patients focused on muscle catabolism, although recently the CNS, especially the hypothalamus, has been shown to be an important mediator of the process [106,107]. The hypothalamic arcuate nucleus contains neuropeptide Y (NPY)/agouti-related regulatory peptide (AgRP) neurons that increase appetite and reduce energy consumption, as well as pro-opiomelanocortin (POMC) neurons that suppress appetite and increase energy consumption. POMC produces α-melanocyte-stimulating hormone (α-MSH), which acts as an agonist for melanocortin-4 receptors (MC4R) to suppress appetite. NPY and AgRP act to increase appetite via NPY receptors and MC4R, respectively. AgRP is an inverse agonist of MC4R that acts in opposition to α-MSH to increase appetite [108]. The gastrointestinal tract secretes appetite-stimulating ghrelin, appetite-suppressing peptide YY, cholecystokinin, and glucagon-like peptide 1, and this information on the regulation of eating is transmitted from the vagus nerve afferent pathway to the hypothalamus via the medulla oblongata NTS. Leptin and insulin, which are secreted in proportion to the body fat mass, also send information about energy reserves to the hypothalamus and are involved in the long-term regulation of eating. Inflammation of the hypothalamus directly regulates eating behavior by activating appetite-suppressive pathways and inhibiting appetite-promoting pathways and is also involved in a variety of metabolic outcomes, including energy consumption, carbohydrate/fat/protein use, blood glucose levels, and insulin sensitivity [109]. Patients with cancer cachexia show increased susceptibility to leptin and reduced sensitivity to ghrelin [110].

#### 2.3.4. Dysphagia Due to Loss of Skeletal Muscle

A decrease in muscle mass and function contributes to dysphagia. In a systematic review by Zhao et al. [111], the odds ratio between primary sarcopenia and dysphagia was 4.06. The mechanism of dysphagia due to primary sarcopenia is similar to that of muscle-related secondary sarcopenia, which is associated with inactivity, malnutrition, and post-disease swallowing [112]. Generalized muscle loss is likely to reduce the functions of the muscles involved in swallowing, including the mimetic muscles around the mouth, masticatory muscle group, lingual muscle group, suprahyoid muscle group, infrahyoid muscle group, soft palate muscle group, and pharyngeal muscle group. Skeletal muscle mass has been associated with severe dysphagia in patients with cancer [113]. Imaging studies using ultrasound and other methods have observed atrophy of the tongue, thinning of the pharyngeal wall, and decreased muscle mass in the anteroventral hyoid muscles, digastric muscle, and temporal muscle [114,115]. In particular, the cross-sectional area of the geniohyoid muscle is associated with tongue pressure, jaw-opening strength, and duration of swallowing sounds [116,117] and correlates with skeletal muscle mass and nutritional status [118,119]. Tongue pressure is also used to diagnose sarcopenic dysphagia, with a cutoff value of <20 kPa [114]. Sarcopenic dysphagia in patients with cancer may progress latently even before the onset of cancer cachexia. It is important to prevent, treat, and promote recovery from sarcopenic dysphagia with physical rehabilitation, dysphagia rehabilitation, and nutritional support based on appropriate assessments [120,121,122].

#### 2.3.5. Multimodal Intervention for Advanced Cancer Management

Progressive skeletal muscle loss is characterized by a negative protein and energy balance driven by a variable combination of reduced food intake and abnormal metabolism. The underlying factors contributing to reduced food intake should be assessed. These include a decreased central drive to eat, chemosensory disturbances (for example, in taste and smell), decreased upper gastrointestinal motility (for example, early satiety and nausea), and distal tract dysmotility (after treatment of constipation) [97]. In summary, various combinations of poor appetite or reduced food intake and abnormal metabolism are factors that cause a decrease in skeletal muscle mass. One cause of dysphagia in cancer patients is the loss of the swallowing muscles accompanying generalized skeletal muscle loss [113]. As multiple mechanisms are involved in the development and progression of weakness of swallowing-related muscles, there is no guarantee that treatments that focus on a single factor will be effective or safe.

Exercise may ameliorate metabolic abnormalities in cancer by improving insulin resistance, hyperlipidemia, mitochondrial function, and inflammation, and by promoting muscle mass gain [123]. Therefore, exercise therapy for dysphagia in patients with advanced cancer can be divided into two types: exercise programs for the muscles involved in swallowing and exercise training to improve the physical function.

Carmi-gnani et al. [124] performed swallowing exercises for patients with advanced head and neck cancer prior to radiation therapy or chemoradiation. The results suggest that the ability to swallow can be significantly improved, which has a positive impact on the patient’s quality of life. There is also a study on the long-term swallowing function of patients with advanced head and neck cancer when chemoradiation therapy and swal-lowing exercises are administered simultaneously. This study concluded that dysphagia was minimal six years after chemoradiotherapy [125].

In a study of exercise training to improve physical function in advanced cancer patients receiving palliative care, there were beneficial effects on symptoms, such as pain, fatigue, depression, anxiety, sleepiness, happiness, and appetite [126]. Improved quality of life and reduced fatigue were observed [127]. In addition, a study of physical therapy in advanced cancer patients with less than two years of life showed improved physical performance [128].

The direct benefit of exercise on dysphagia due to the loss of skeletal muscle mass in patients with cancer has not been determined. However, Nagano et al. [129] reported that swallowing function improved in elderly hospitalized patients with sarcopenia after physical intervention and nutritional management. This suggests that physical exercise may be effective in treating dysphagia caused by skeletal muscle loss.

It is well known that nutritional therapy plays an important role in not only cancer survivors but also patients with advanced cancer. To maintain or improve food intake, alleviate metabolic abnormalities, and maintain skeletal muscle, parameters relevant to cancer patients should be monitored regularly, and caloric and protein intake should be increased. In addition, a combination of pharmacological treatments, such as drugs that modulate cytokine action, appetite regulation, and metabolic targets, can have additive or synergistic effects. Solheim et al. [130] conducted a study of multimodal interventions (nutritional supplements, nutritional counseling, physical therapy, and anti-inflammatory drugs) for cancer cachexia in patients with refractory lung or pancreatic cancer. As a result, their weight stabilized. A phase III trial is currently underway [131].

Cancer patients often experience weight loss accompanied by anorexia and other debilitating symptoms that affect their daily lives. Psychosocial strategies for patients with advanced cancer include reducing weight and diet-related distress in patients and their caregivers and moving toward conscious control over eating. These help to maintain the willingness and ability to eat, even in the absence of appetite, if there is no nausea or vomiting [132,133]. In doing so, it may support multimodal therapy intake and compliance.

## 3. Conclusions

Intensive treatment and a medical care team are indispensable components of modern cancer treatment. Historically, advances in cancer treatment have occurred primarily through the introduction of new revolutionary treatments. In recent years, patient-centered therapeutic approaches have permeated cancer care. The treatment options have diversified based on individual characteristics, such as performance status, pharmacotherapeutic or dynamic genetic background, and social and environmental factors. This extends the duration of treatment and survival of cancer patients, which makes it necessary to consider the long-term risks of dysphagia.

Indeed, dysphagia in cancer patients is associated with factors, such as tumor effects, surgery, chemotherapy, and radiation therapy, and may be unavoidable. However, as it is associated with multiple mechanisms, it may be preventable or ameliorated by treatment. These include respiratory swallowing training, ion channel agonist supplementation, aromatherapy, exercise therapy, compensatory approaches, nutritional support, and psychosocial interventions.

The diagnosis and treatment of cancer require comprehensive screening and assessment, including the possibility of swallowing dysfunction. To devise treatment plans for cancer patients, a team of various specialists, including doctors, nurses, psychologists, dietitians, physical therapists, occupational therapists, and speech therapists, who have specialized knowledge and skills related to surgery, radiation therapy, pharmacotherapy, pathological diagnosis, and palliative care, need to create a multifaceted and holistic treatment process from a long-term perspective.

## Figures and Tables

**Table 1:** The taste receptors that detect taste qualities.

Taste	Taste Receptors	
Sweet	G protein-coupled receptors (GPCRs)	Taste 1 receptor member 2 and 3 (TAS1R2 + TAS1R3)
Umami		Taste 1 receptor member 1 and 3 (TAS1R1 + TAS1R3)
Bitter		Taste 2 receptor (TAS2R)
Salt	Ion channel receptors	Epithelial sodium channel (ENaC)
Sour		Transient receptor potential vanilloid 1 (TRPV1) Acid-sensing ion channel 3 (ASIC 3)
Pungency		TRPV1 Transient receptor potential ankyrin 1 (TRPA1)

Note: Transient receptor potential channel M5 (TRPM5) is a downstream effector of G protein-coupled taste receptors.

**Table 2:** Antineoplastics that can cause dysgeusia.

Class	Name
Alkylating agents	Thiotepa (42.1%), Streptozocin (22.7%), Busulfan (<5~20%), Trabectedin (<5~20%), Bendamustine (≥10%), Temozolomide (<10%), and Cyclophosphamide (<0.1~5%)
Antimetabolites	Capecitabine (10.2~27.3%), Gemcitabine (<1~10%), Nelarabine (<1~10%), Doxifluridine (≥5%), Trifluridine and tipiracil hydrochloride (<5%), Pemetrexed disodium (<5%), Fluorouracil (<0.1%), Tegafur and uracil (<0.1~5%), Tegafur (<0.1%), Fludarabine (Frequency unknown), Methotrexate (Frequency unknown), Tegafur gimeracil, and Oteracil potassium (Frequency unknown)
Plant alkaloids	
Microtubule inhibitors (taxanes)	Eribulin mesylate (33.3%), Vincristine (25.5%), Cabazitaxel (<5~20%), Paclitaxel (<5~20%), Vinblastine (≥5%), Docetaxel (<5%), Vinorelbine (<5%), and Vindesine (<5%)
Topoisomerase inhibitors	Nogitecan hydrochloride (<5%,40.0% in combination with Cisplatin), Sobuzoxane (<0.1~5%), Pirarubicin (<0.1~5%), Etoposide (<1%), Mitoxantrone (<0.1%), and Irinotecan (Frequency unknown)
Anthracycline antibiotics	Amrubicin (<5~30%) and Doxorubicin (<1%)
Platinum compounds	Cisplatin (<10%), Oxaliplatin (≥5%), Nedaplatin (<0.1~5%), and Carboplatin (Frequency unknown)
Hormonal agents	Apalutamide (<5%), Estramustine (≥5%), Exemestane (<0.1~5%), Enzalutamide (<1~5%), Abiraterone acetate (<1%), Flutamide (<1%), Letrozole (<1%), and Anastrozole (<0.1~1%)
Molecular targeted agents	Entrectinib(42.3%), Sunitinib malate (37.5~47%), Cabozantinib (31~41%), Alectinib (23.4%), Crizotinib(20.4%), Axitinib (11.6%), Pazopanib hydrochloride (<5~30%), Abemaciclib (<5~20%), Everolimus (≥10%), Olaparib (≥10%), Quizartinib (<5~10%), Gilteritinib (<5~10%), Niraparib (<5~10%), Trastuzumab (<2~10%), Obinutuzumab (<2~10%), Dacomitinib (<1~10%), Sorafenib (<1~10%), Pembrolizumab (<1~10%), Afatinib (<1~10%), Osimertinib mesylate (<1~10%), Vandetanib (<1~10%), Panitumumab (<0.5~10%), Brentuximab vedotin (<5~10%), Azacitidine (<10%), Trastuzumab deruxtecan (<10%), Palbociclib (<10%), Ponatinib (<10%), Dasatinib (<10%), Vemurafenib (≥5%), Binimetinib (≥5%), Pertuzumab (≥5%), Lapatinib ditosylate (<1~10%), Lenvatinib mesylate (<5~10%), Trastuzumab emtansine (≥5%), Avelumab (≥5%), Panobinostat (≥5%), Inotuzumab ozogamicin (<2~5%), Erlotinib hydrochloride (<1~5%), Nivolumab (<1~5%), Atezolizumab (<1~5%), Tepotinib (<1~5%), Carfilzomib (<1%~5%), Gemtuzumab ozogamicin (<5%), Brigatinib (<5%), Ixazomib (<5%), Ipilimumab (<5%), Encorafenib (<5%), Mogamulizumab (<5%), Trastuzumab (<2%), Nilotinib (≥1%), Imatinib mesylate (Frequency unknown), Alemtuzumab (Frequency unknown), and Tamoxifen citrate (Frequency unknown)
Immunosuppressants	Lenalidomide hydrate (≥ 5%), Sirolimus (<1~5%), and Pomalidomide (<5%)
Others	Borofalan-10B (Boron drug (71.4%) Vorinostat (14.1~23.3%), Romidepsin (≥10%), Tirabrutinib hydrochloride (<5%), Bevacizumab (<1~5%), Temsirolimus (<5%), Necitumumab (<5%), Forodesine hydrochloride (<5%), Bortezomib (<5%) Thalidomide (≥5%), and Arsenic trioxide (<5%)

Note: The numbers in parentheses are the frequency of occurrence of dysgeusia described in the Japanese medical product package insert.
